# Three-Dimensional ZnO Hierarchical Nanostructures: Solution Phase Synthesis and Applications

**DOI:** 10.3390/ma10111304

**Published:** 2017-11-13

**Authors:** Xiaoliang Wang, Mashkoor Ahmad, Hongyu Sun

**Affiliations:** 1 College of Science, Hebei University of Science and Technology, Shijiazhuang 050018, China; 2 Nanomaterials Research Group, Physics Division, Pakistan Institute of Nuclear Science and Technology, P.O. Nilore, Islamabad 44000, Pakistan; mashkoorahmad2003@yahoo.com; 3 Department of Micro- and Nanotechnology, Technical University of Denmark, 2800 Kongens Lyngby, Denmark

**Keywords:** zinc oxide, hierarchical nanostructures, solution phase synthesis, photocatalysis, field emission, sensor, lithium ion batteries

## Abstract

Zinc oxide (ZnO) nanostructures have been studied extensively in the past 20 years due to their novel electronic, photonic, mechanical and electrochemical properties. Recently, more attention has been paid to assemble nanoscale building blocks into three-dimensional (3D) complex hierarchical structures, which not only inherit the excellent properties of the single building blocks but also provide potential applications in the bottom-up fabrication of functional devices. This review article focuses on 3D ZnO hierarchical nanostructures, and summarizes major advances in the solution phase synthesis, applications in environment, and electrical/electrochemical devices. We present the principles and growth mechanisms of ZnO nanostructures via different solution methods, with an emphasis on rational control of the morphology and assembly. We then discuss the applications of 3D ZnO hierarchical nanostructures in photocatalysis, field emission, electrochemical sensor, and lithium ion batteries. Throughout the discussion, the relationship between the device performance and the microstructures of 3D ZnO hierarchical nanostructures will be highlighted. This review concludes with a personal perspective on the current challenges and future research.

## 1. Introduction

Advanced nanomaterials, which are earth abundant and environmentally compatible, show the potential to solve the serious energy and environment problems. As an important and widely used wide bandgap (3.0–3.2 eV) oxide semiconductor, ZnO shows unique physical and chemical properties [1]. The applications of ZnO materials range from room temperature nanolasers, nanogenerators, solar cells, lithium ion batteries and photocatalysts.

**Figure 1:**
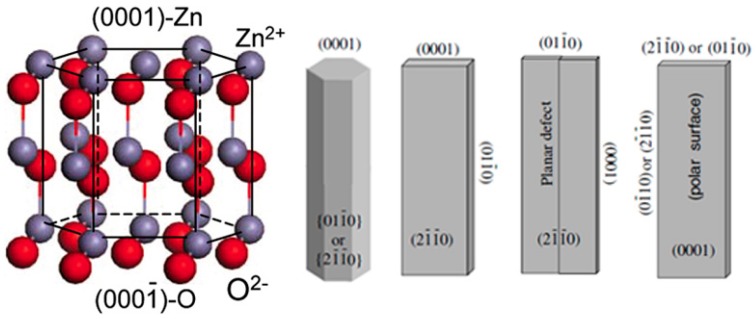
Crystal structure model of wurtzite ZnO (reprinted from [5] with permission, Copyright—Elsevier B.V., 2004), and typical morphologies of 1D ZnO nanostructures with exposed facets (reprinted from [2] with permission, Copyright—Elsevier B.V., 2009).

ZnO crystal shows the stable structure as hexagonal wurtzite under the condition of normal temperature and atmospheric pressure. The crystal structure of ZnO can be viewed as a number of alternating planes composed of tetrahedrally coordinated oxygen and zinc ions stacked alternately along the [0001] direction (Figure 1). There are two important structural characteristics in wurtzite ZnO, i.e., the absence of inversion symmetry of the positive and negative charge centers and polar surfaces, which are the origin of piezoelectric properties and unique growth behaviors in the synthesized ZnO nanostructures. The most typical polar surface in ZnO structure is the basal plane (Figure 1), in which positively charged Zn-(0001) and negatively charged O-(000-1) polar surfaces are produced. These net charges on the polar surfaces are ionic charges and non-mobile. The distribution of surface charges is responsible for the minimizing the electrostatic energy of the system, which is also one of the important driving forces for growing nanostructures with the domination of polar surfaces [2]. In principle, the equilibrium morphology of a crystal is determined by the standard Wulff construction, which depends on the relaxation energies [3]. For ZnO crystals, the kinetic parameters vary with different crystal planes and growth direction due to different relaxation energies, which are emphasized under a given growth condition [4]. Therefore, from the viewpoint of decreasing the system total energy, a ZnO crystallite will commonly develop into a three-dimensional morphology with well-defined and low-index crystallographic faces (Figure 1). In addition, the surface energy can be modified by selective adsorption of additives or surfactants on specific planes, and the morphology can be controlled accordingly by adding suitable agents during the synthesis.

Inspired by the size- or morphology-dependent properties or device performances, numerous efforts have been devoted to the synthesis of ZnO nanostructures with 1D morphologies, such as nanowires, nanobelts, nanorings, nanohelices and so on [2,5,6]. Recently, more attention has been paid to assembling low-dimensional nano-sized building blocks into three-dimensional (3D) complex hierarchical structures [7]. Compared to mono-morphological structures, 3D ZnO hierarchical structures usually exhibit high surface to volume ratios, a large accessible surface area and better permeability. In addition, the hierarchical structures can increase the number of light traveling paths and thereby facilitate light absorption. Finally, such 3D hierarchical structures not only inherit the excellent properties of the single nano-sized building blocks but also provide potential applications in the bottom-up fabrication of functional devices including photocatalysts, sensors and drug release systems [8]. It is, therefore, important to develop facile approaches to synthesize 3D hierarchical structures with controlled fashion.

**Figure 2:**
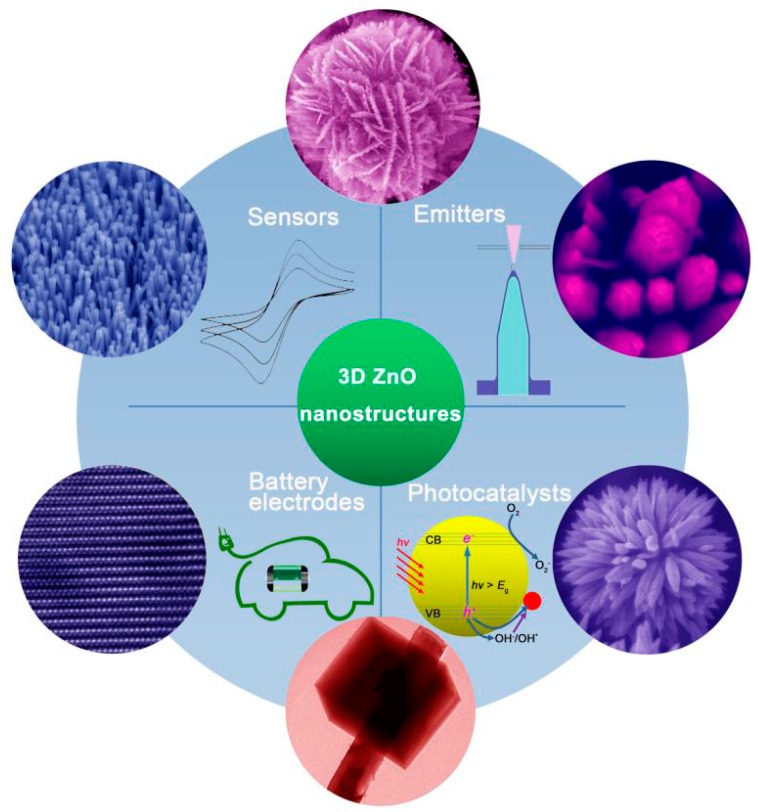
Typical 3D ZnO hierarchical nanostructures and their applications as photocatalysts, field electron emitters, electrochemical sensors, and electrodes for batteries.

Herein, we summarize the most recent progress in the synthesis of 3D ZnO hierarchical nanostructures by using solution phase routes, and discuss the related applications. Firstly, typical solution phase synthesis methods towards 3D ZnO hierarchical nanostructures are reviewed, including direct precipitation, microemulsions, hydrothermal/solvothermal, sol-gel, electrochemical deposition, and chemical bath deposition. The basic principles of the synthesis and main factors that influence the structure and morphology of the products are analyzed. Then, different applications based on the 3D hierarchical architectures are discussed in the context of photocatalysis, field emission, electrochemical sensors, and electrodes for lithium ion batteries (Figure 2). Finally, current challenges and future outlooks of the synthesis and applications of 3D ZnO hierarchical nanostructures are briefly outlined.

## 2. Solution Phase Synthesis of 3D ZnO Hierarchical Nanostructures

The above discussion shows that the hierarchical assembly in ZnO nanostructures is related to the electronic/optical properties and thus a wide range of potential applications. So far, many synthesis strategies based on physical (physical and chemical vapor deposition, laser ablation, ball milling, lithographic, etc.), chemical (gas phase reaction, various solution phase synthesis), or biological methods have been well established to obtain 3D ZnO hierarchical nanostructures [9]. Comparing to other methods, solution phase route shows unique advantages, such as low cost (low in energy consumption and equipment costs), scalability, and ease of handling. Most of the solution phase reactions occur under mild condition with a relatively low temperature (<200 °C). Therefore, solution phase synthesis has attracted increasing interest. Typical solution phase synthesis includes precipitation, microemulsions, hydrothermal/solvothermal, sol-gel, electrochemical deposition, chemical bath deposition, and so on. There are several excellent reviews describing the synthesis of ZnO nanostructures [10,11,12,13]. In this paper, we mainly focus on ZnO hierarchical nanostructures synthesized by different solution phase methods.

### 2.1. Precipitation

**Figure 3:**
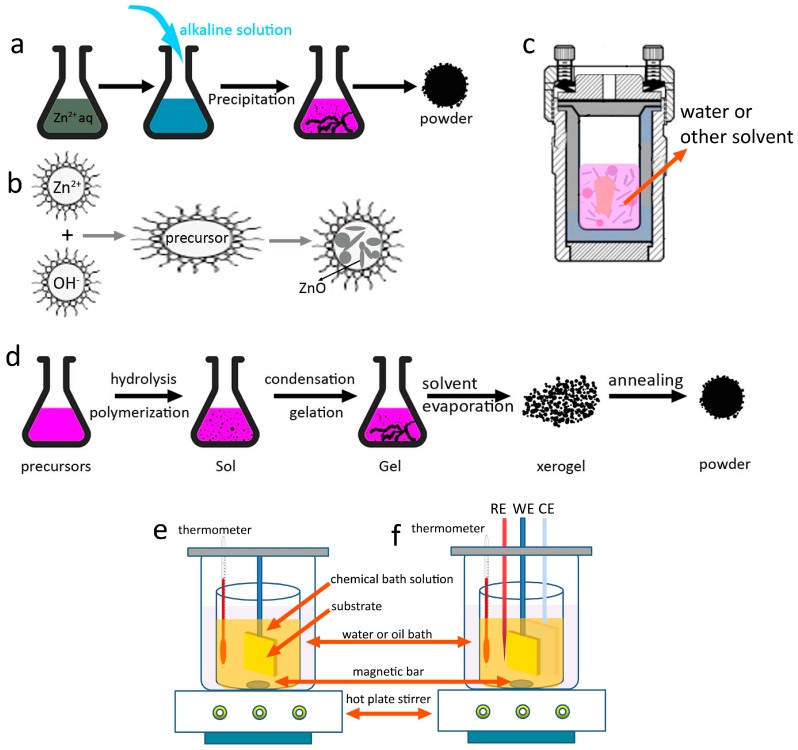
Typical solution phase methods for the synthesis of 3D ZnO hierarchical nanostructures. (**a**) Precipitation; (**b**) microemulsions; (**c**) hydrothermal/solvothermal; (**d**) sol-gel; (**e**) chemical bath deposition; and (**f**) electrochemical deposition.

In a typical precipitation process, different kinds of alkalis (NaOH, KOH, ammonium, urea, hexamethylene tetramine (HMT), etc.) and zinc sources (zinc salts, zinc foil, etc.) are used (Figure 3a). The synthesis starts with a reaction between zinc and hydroxide ions followed by the process of aggregation. A resultant precipitate is collected by filtration or centrifugation. The morphology and assembly of the ZnO products can be controlled by adjusting the reaction conditions, including the concentration of reacted solutions, temperature, time, and additives to tune the reaction steps [14]. Kołodziejczak-Radzimska and co-workers [15] optimized the precipitation conditions that would ensure getting the uniform particles of ZnO with the minimum diameter. Sepulveda-Guzman et al. [16] synthesized submicron ZnO arrays by a simple one-step aqueous precipitation method with zinc nitrate (Zn(NO_3_)_2_) and sodium hydroxide (NaOH) as the reagents. The effect of reaction temperature on the morphology change was studied. Snowflake-like and flower-like morphologies were obtained at 60 °C and 70 °C. The ZnO arrays are formed through self-aggregation process, and that such an oriented aggregation is enhanced by increasing the reaction temperature. In another work, Oliveira and co-workers [17] systematically investigated the influence of zinc salts (Zn(NO_3_)_2_·6H_2_O, zinc sulfate (ZnSO_4_·7H_2_O)), pH value, temperature, additives (sodium sulfate, sodium dodecyl sulfate) on the final ZnO morphology and size. The results show the importance of nucleation of nanometric primary particles followed by oriented aggregation to produce uniform submicrometric particles. Recently, López et al. [18] reported an interesting work on exploring the synthesis of ZnO by employing zinc sources leached from alkaline batteries, paving a new way to recycling useful nanostructures from various chemical wastes.

**Figure 4:**
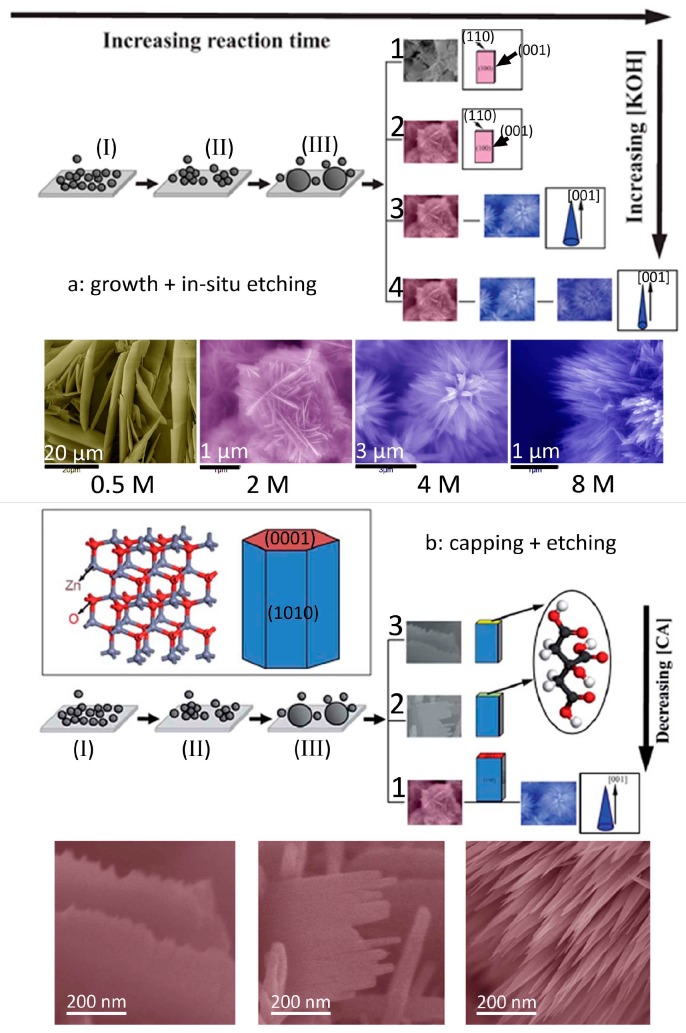
Schematic illustration of the formation process of ZnO 3D hierarchical structures via the combination of (**a**) growth and in situ etching (reprinted from [8] with permission, Copyright—The Royal Society of Chemistry, 2012); (**b**) capping and etching (reprinted from [23] with permission, Copyright—The Royal Society of Chemistry, 2015).

It should be noted that ZnO is a typical amphoteric oxide, which can be etched in either an acid or alkali environment. The reaction between the zinc ions and alkaline ions results in the formation of ZnO crystals. Meanwhile, the newly formed ZnO further reacts with alkaline in growth solution until an equilibrium is achieved. Thus, tuning the precipitation (growth) and in situ etching provides a facile approach to control the morphology of nanocrystals [19,20,21]. For example, Xi et al. [19] fabricated large-scale arrays of highly oriented single-crystal ZnO nanotubes by an in situ chemical etching of the ZnO nanorods. Yang et al. [20] have demonstrated a novel anisotropic etching methodology for the synthesis of complex Ag nanoparticles shapes by controlling the concentrations of etching solution, and highlighted their important applications as highly sensitive surface enhanced Raman spectroscopy substrates. In our previous studies [8], we reported a simple precipitation to synthesize ZnO 3D hierarchical structures by using Zn(NO_3_)_2_·6H_2_O, Zn foil, and KOH as the zinc source and alkalis. Importantly, the morphology of the hierarchical structures can be simply selected by changing the KOH concentrations. The morphology of the products are sheet, flower-like microsphere assembled by nanosheets, flower-like microsphere assembled by nanoneedles, and flower-like microsphere assembled by thinner nanoneedles when the concentration of KOH increased from 0.5 to 2, 4, and 8 M, respectively (Figure 4a). A possible formation mechanism based on preferential etching of KOH along the [001] direction of ZnO sheets was proposed (Figure 4a). The growth or/and etching can be further controlled by adding suitable capping agents that prefer to absorb on specific crystal planes, making it possible to have fine control over the morphology. Tian et al. [22] demonstrated that citrate ions can selectively adsorb on the (001) surfaces of ZnO crystals and thus inhibit the growth along [001] orientation. Based on this knowledge, they synthesized large arrays of oriented ZnO nanorods with controlled aspect ratios and a series of complex morphologies. In our studies, we combined the selective capping (citrate ions) and etching (KOH) together to capture the intermediate of the morphology change, and proposed a possible formation mechanism based on capping–etching competitive interactions (Figure 4b) [23]. The strategy can also be applied to the synthesis and modification of other materials by carefully selecting suitable capping agents and etchants.

### 2.2. Microemulsions

**Figure 5:**
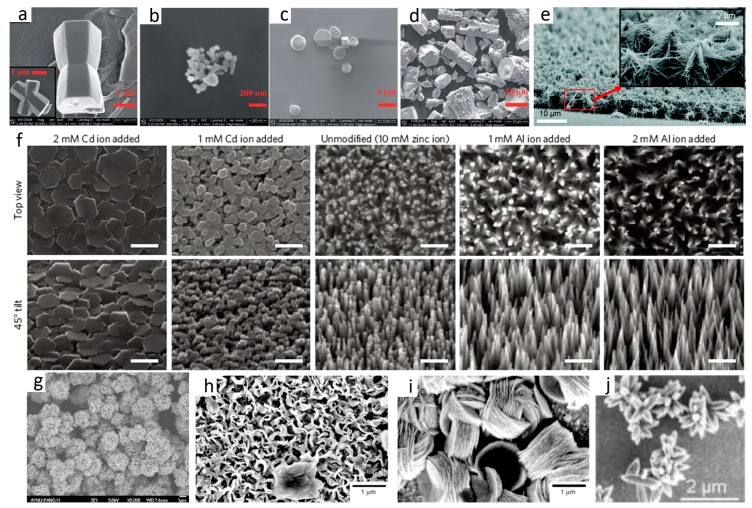
Typical 3D ZnO hierarchical nanostructures synthesized by solution phase methods: (**a**–**d**) microemulsion process (reprinted from [25] with permission, Copyright—The Royal Society of Chemistry, 2012); (**e**) repetitive hydrothermal (reprinted from [35] with permission, open access, American Chemical Society, 2011); (**f**) ion-mediated hydrothermal (scale bars = 500 nm, reprinted from [36] with permission, Copyright—Macmillan Publishers Limited, 2011); (**g**) sol-gel method (reprinted from [39] with permission, Copyright—Elsevier Ltd and Techna Group S.r.l., 2013); (**h**,**i**) electrochemical deposition (reprinted from [49] with permission, Copyright—The Owner Societies, 2011); and (**j**) chemical bath deposition method (reprinted from [51] with permission, Copyright—American Chemical Society, 2015).

Materials synthesis via microemulsion is occurred in a stable mixed solution that contains water, oil, and surfactant. Depending on the properties of immiscible liquid–liquid interface, the formation of microemulsion can be in the form of oil-swollen micelles dispersed in water (oil-in-water, or O/W) or the reverse case, i.e., water swollen micelles dispersed in oil (water-in-oil, W/O). The two configurations are usually named as microemulsion and reverse microemulsion, respectively [24]. Figure 3b shows a typical process for the synthesis of ZnO via microemulsion method. The typical size of the microemulsion or reverse microemulsion is smaller than 100 nm; therefore, the microemulsions can be used as nanoreactors for materials synthesis, which provides a bottom-up route to control the size and morphology by adjusting the microemulsion properties. By studying the phase diagrams of oil-water-surfactant system containing toluene, zinc acetate solution, cetyltrimethylammonium bromide and butanol, Lin et al. [25] obtained the desired size and shape of ZnO particles (Figure 5a–d). This study demonstrates the importance of investigating the intrinsic properties of this multi-liquid-phase system. Jesionowski et al. [26] made emulsion that was composed of cyclohexane as the organic phase, zinc acetate as the water phase and appropriate emulsifiers. The obtained ZnO showed a narrow particle size distribution and large specific surface area. In their further work [27], they synthesized a series of ZnO materials in a similar emulsion system. ZnO structures with the morphologies of solids, ellipsoids, rods and flakes were successfully obtained by applying modifications of the ZnO precipitation process.

### 2.3. Hydrothermal and Solvothermal

Hydrothermal synthesis refers to the materials synthesis by chemical reactions of substances in a sealed heated aqueous solution above ambient temperature and pressure, while solvothermal is very similar to the hydrothermal route except the precursor solution is usually non-aqueous (Figure 3c) [28,29,30,31]. Well control over the hydrothermal/solvothermal synthetic conditions is a key to the synthesis of ZnO nanomaterials with defined structure, morphology, composition, and assembly. Typical control experimental parameters include reagents, solvent, additives, filling degree, temperature, time, and so on [32,33]. In addition, substrate is an important and interesting parameter that can affect the ZnO morphology, especially the aligned ZnO nanostructures. In a study, ZnO nanostructures on Zn foil was synthesized by hydrothermal synthesis [34]. The morphology and assembly of ZnO arrays are dependent on the solvent properties. Specifically, ZnO nanorod arrays and randomly scattered nanorods are obtained in the mixed solvent containing ammonia aqueous solution (1%) and pure water, and pure water system, respectively. Moreover, repetitive hydrothermal or solvothermal treatment can yield more complex and hierarchical configurations. By employing this simple strategy, Ko et al. [35] synthesized high density and long branched tree-like ZnO nanoforests (Figure 5e). In a report of Joo et al. [36], single-crystalline ZnO nanowires were grown on substrates with zinc oxide seed layers in aqueous solutions by hydrothermal method. The effect of specific additives on the ZnO morphology was studied in detail. The results show that the addition of positively charged complex ions (Cd, Cu, Mg, Ca) and negatively charged complexes (Al, In, Ga) promote low and high aspect ratio growth of ZnO nanostructures (Figure 5f). They demonstrated that face-selective electrostatic crystal growth inhibition mechanism governed this selective synthesis. Wysokowski et al. [37] used β-Chitinous scaffolds as a template during the hydrothermal synthesis of ZnO under mild conditions (70 °C). The obtained samples showed unique film-like morphology, which showed good antibacterial properties against Gram-positive bacteria. Very recently, Van Thuan and Kim et al. [38] synthesized oval-shaped graphene/ZnO quantum hybrids by employing a facile chemical-hydrothermal method. The samples exhibited excellent catalytic properties for the selective reduction of nitroarenes.

### 2.4. Sol-Gel Processing

The sol-gel process contains the formation of solid material from a solution by using a sol or a gel as an intermediate step. The synthesis of metal oxide materials often involves controlled hydrolysis and condensation of the alkoxide precursors or salts. Figure 3d illustrates the main steps of preparation of metal oxides powder by the sol-gel process: (i) preparation of the precursor solution; (ii) hydrolysis of the molecular precursor and polymerization via successive bimolecular additions of ions, forming oxo-, hydroxyl, or aquabridges; (iii) condensation by dehydration; (iv) solvent evaporation and organic compounds removal to form xerogel; and (v) heat treatment of the xerogel to form powers. Properties of the final products, including the particle size, surface area, crystallinity, and agglomeration, are highly dependent on the reaction parameters, especially the precursors, solvents, additives, evaporation, drying, and post-treatment conditions [39] (Figure 5g). Tseng et al. [40] employed the sol-gel process to synthesize ZnO polycrystalline nanostructures using Zn(CH_3_COO)_2_·2H_2_O as the zinc solute and different alcohols as solvents (glycol, glycerol, and diethylene glycol). The morphology of the final ZnO was fiber, rhombic flakes, and spherical particles. The formation of thorn-like ZnO nanostructures in the sol-gel process was reported by Khan and co-workers [41]. They further modified this method by mechanical stirring during the sol generation, and found the agitation speed was a critical value in determining the size and aspect ratio of the particles. Higher stirring speed is favorable for anisotropic growth of ZnO nanoparticles.

### 2.5. Electrochemical and Chemical Bath Deposition

Electrochemical deposition (ECD) and chemical bath deposition (CBD) methods are facile and can produce materials or nanostructures that cannot be obtained by other deposition methods. The CBD process only requires suitable solution containers and substrate mounting devices (Figure 3e), while for the ECD method, additional power supplies, electrodes (counter electrode/CE, reference electrode/RE) are necessary, and the substrate (working electrode/WE) must be conductive (Figure 3f). More importantly, the morphology and orientation of the deposited samples can be tuned by controlling the reaction thermodynamics and kinetics, including solution properties, additives, substrate, temperature, and electrochemical parameters (applied potential, current density, etc.) [42,43,44,45,46,47].

The synthesis of ZnO nanostructures via the ECD process includes the reduction of precursor at the electrode, the supersaturation at the vicinity of the electrode, and subsequent precipitation. Theoretically, factors that affect any step should be considered to achieve the good control of final structures. In this regard, Illy et al. [48] systematically studied the effect of various experimental parameters (electrolyte concentration, pH value, reaction temperature, and overpotential) on the morphology, thickness, transparency, roughness and crystallographic orientation of the ZnO materials. They found that ZnO nanostructures with (002) preferential orientation and controlled thickness can be grown by using optimized parameters, which are important for organic photovoltaic applications. Different additives can either interact with the ions in the electrolyte, absorb on specific sites on the deposited ZnO structure, or change the electrolyte itself (conductivity, viscosity, etc.), resulting in a different deposition pathways and thus final products. In a study by Oekermann and co-workers [49], the addition of water soluble tetrasulfonated metallophthalocyanines (TSPcMt), in which Mt = Zn(II), Al(III)[OH] or Si(IV)[OH], in the electrolyte containing zinc nitrate yields completely different morphology and assembly, which is ascribed to the preferential adsorption of the additive molecules onto the crystal planes of ZnO (Figure 5h,i).

Synthesis of ZnO nanostructures via CBD is based on a direct chemical reaction involving dissolved zinc ions and oxygen precursors in the solution. Different from ECD where the deposition only occurs on the conductive substrate, the growth of ZnO in CBD process can take place either in the solution or on the substrate surface. The morphology and assembly of ZnO products can also be controlled by the solution, additives, and so on [50]. Moreover, patterned or flexible ZnO hierarchical nanostructures can be obtained by applying corresponding substrates [51] (Figure 5j).

The above-mentioned solution phase synthesis methods have their own advantages and disadvantages. Table 1 summarizes and compares the methods for the synthesis of 3D ZnO hierarchical nanostructures. In addition, the different solution phase methods can be combined together or with other treatments, such as microwave heating, sonochemistry, etc., to achieve even more complex and interesting hierarchical nanostructures with useful applications. For example, sol-gel processing or microemulsions are often employed with hydrothermal/solvothermal treatment to prepare various nanostructures [52]. The pre-synthesized ZnO nanostructures via hydrothermal or ECD methods can be further etched to form needle or tube arrays [53]. The assembly of ZnO architectures can be tuned by means of ECD via deformation and coalescence of soft colloidal templates in reverse microemulsion [54]. It is worth noting that a wide range of ZnO hierarchical nanostructures can also be synthesized from different biomass, such as microorganisms, enzymes, bacteria, and plant extracts, which are eco-friendly alternatives compared to the conventional synthesis methods [55,56,57]. In a word, solution phase synthesis provides plenty of room to control and optimize hierarchical ZnO architectures for diverse applications.

## 3. Applications of 3D ZnO Hierarchical Nanostructures

3D ZnO hierarchical nanostructures show unique advantages of high surface area, porous structures, and synergistic interactions of the constituted nano building blocks. Therefore, 3D ZnO nanostructures possess improved physical/chemical properties, such as enhanced light harvesting, increased reaction sites, and improved electron and ion transportation, which are highly needed for optical, electrical, and electrochemical applications. In this paper, we will review the most recent progress in the research activities on 3D ZnO hierarchical nanostructures used for photocatalysis, field emission, electrochemical sensors, and electrodes for lithium ion batteries.

### 3.1. Photocatalysis

**Figure 6:**
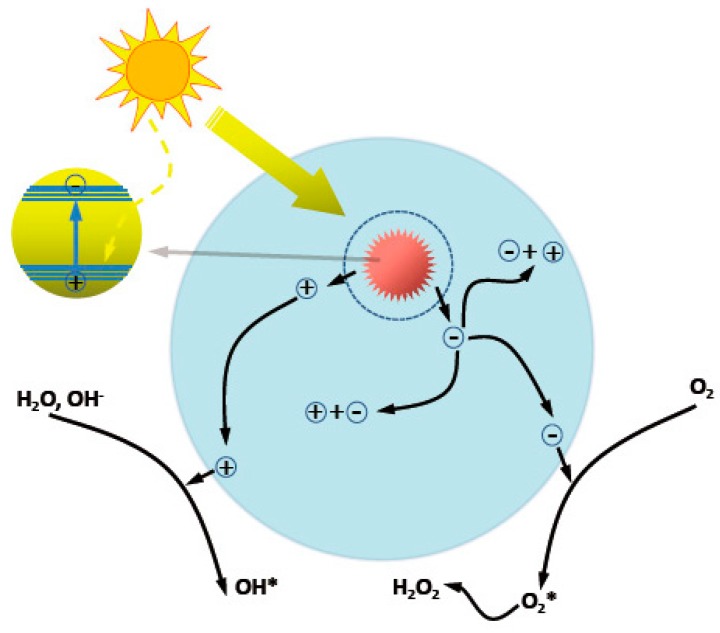
Schematic illustration on the photocatalytic processes in ZnO.

ZnO nanostructures have attracted much attention to the fields of photocatalysis, including photocatalytic degradation of organic contaminants, photocatalytic water splitting, and so on, due to the notable merits such as nontoxicity, biological compatibility, and universality. Typical steps involved in heterogeneous photocatalysis process are as follows (Figure 6) [58]: (1) light absorption; (2) the generation and separation of photoexcited electrons and holes; (3) the migration, transport and recombination of carriers; and (4) surface electrocatalytic reduction and oxidation reactions. The overall catalysis efficiency is related to the cumulative effects of these consecutive steps. For ZnO photocatalysts, the activity is limited by the intrinsic wide bandgap (3.0–3.2 eV) and the high electron–hole recombination rate, which can be tuned by optimizing the structural parameters of the photocatalysts, such as size, morphology, assembly, specific surface area, and the defect density [59]. Compared to the 0D, 1D, or 2D counterparts, and 3D ZnO hierarchical nanostructures show advantages of high surface area and porous structures, enhanced light harvesting, and synergistic effects between the nano building blocks. All of these characters are beneficial for the photocatalysis enhancement.

**Figure 7:**
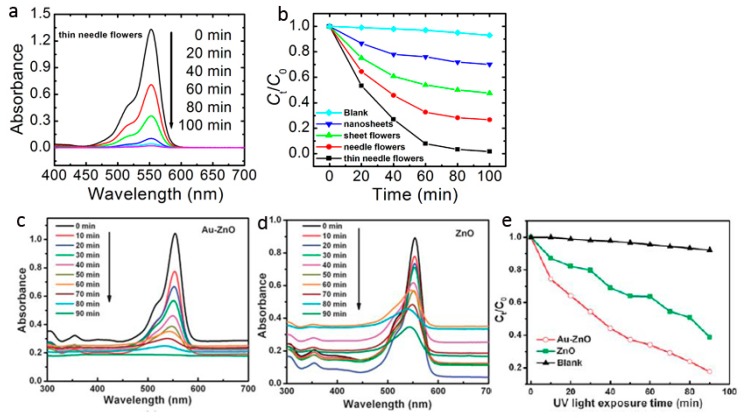
Photocatalytic degradation of RhB via (**a**,**b**) 3D ZnO hierarchical nanostructures with different morphologies (reprinted from [8] with permission, Copyright—The Royal Society of Chemistry, 2012); (**c**–**e**) ZnO needle flowers and Au nanoparticles/ZnO needle flowers composite (reprinted from [65] with permission, Copyright—The Royal Society of Chemistry, 2011).

The comparison of the photodegradation of organic dye Rhodamine B (RhB) under UV-irradiation with different 3D ZnO hierarchical nanostructures yielded by facile solution phase method is shown in (Figure 7a,b) [8]. Under the same experimental conditions, the relative photocatalytic activity is thin needle flowers > needle flowers > sheet flowers > nanosheets. The significant improvement in the photocatalytic activity of the thin needle flowers structure can be attributed to the following reasons: (1) optical quality and special structural features; (2) the large active surface area and interspaces of the flower structure, which facilitate the diffusion and mass transportation of RhB molecules and hydroxyl radicals; (3) the improved efficiency of electron–hole separation. The morphology of ZnO nanostructures dependent photocatalysis is later demonstrated in the degradation of methylene blue in aqueous solution [60]. By coupling the strategies of elemental doping [61], defect engineering [62], modifying the surface with visible light active materials [63] or plasmonic-metal nanostructures (Ag, Pt, Au, etc.) [64], and the photodegradation properties of 3D ZnO hierarchical nanostructures can be further improved. For example, we evaluated photocatalytic performance of ZnO needle flowers and Au nanoparticles/ZnO needle flowers composite (Au/ZnO) by degradating organic dye RhB under UV irradiation (Figure 7c–e) [65]. The pure ZnO showed observable photocatalytic activity but with rather slow kinetics. Only ~60% of the RhB was decomposed within 90 min. In contrast, when the Au/ZnO composites were applied as the photocatalyst, a significant synergistic enhancement effect was observed, i.e., RhB was decomposed thoroughly within 90 min. The Au nanoparticles enhanced photodegradation is also observed in ZnO sheet flowers. In these studies, besides the hierarchical morphology of ZnO nanostructures, the improvement of photocatalytic properties can be ascribed to the presence of noble metal nanoparticles: (1) the light absorption is increased due to the strong surface plasmon resonance of the noble metal nanoparticles; and (2) the efficiency of charge separation of the photo-generated electron–hole pairs is increased due to the strong electronic interaction between strong electronic interaction and ZnO.

The above routes to improve the degradation properties in ZnO hierarchical photocatalysts can also be applied to photosplit water to generate hydrogen. For example, elemental doping and defect engineering are effective to narrow the bandgap of ZnO materials, which results in an extension of light absorption range from UV into visible light range, while generating interface structures by depositing plasmonic noble metals separates photogenerated carriers, improves visible and near-infrared photo-absorption, and thus achieves high-performance photocatalytic hydrogen evolution.

### 3.2. Field Emission

**Figure 8:**
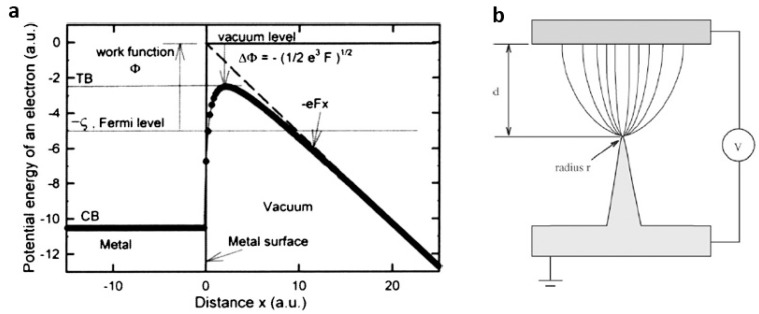
(**a**) Potential energy of an electron near the cathode surface (reprinted from [67] with permission, Copyright American Vacuum Society, 2007); (**b**) illustration of field electron emission from a tip (reprinted from [68] with permission, Copyright—Elsevier B.V., 2004).

Field emission devices show several advantages, such as the resistance to temperature fluctuation and radiation, less power consumption, low thermionic noise, low energy spread, miniature volume and nonlinear, and the exponential current–voltage (I–V) relationship in which a small variation in the voltage results in a large change in the emission current instantaneously [66]. Theoretical calculations show that the external filed induces a decrease of the surface barrier height by a value of ΔФ ~ 3.8 *F*^1/2^ (for Ф in eV and *F* in V/Å) (Figure 8a), and the field is off the order of ~10^9^ V/m. If the emitter surface is sharp configuration as shown in Figure 8b, electrons can be extracted at a considerably lower applied field. The relationship between the field emission current density (*J*) and the applied electric field (*E*) is described by Fowler–Nordheim equation: $J=(A\beta^{2}E^{2}/Ф)\exp\lfloor -B{Ф}^{3/2}{\left(\beta E\right)}^{-1}\rfloor$, where A (1.54 × 10^−6^ A eV V^−2^) and B (6.83 × 10^3^ eV^−3/2^ V μm^−1^) are the first and second F-N constants, Ф is the work function, and *β* is the field enhancement factor, which reflects the magnitude of electric field at the emitting surface. The field enhancement factor *β* can be defined as the ratio of local electric field divided to the applied electric field. Therefore, *β* is a dimensionless quantity. The emission current density at a constant cathode-anode distance is strongly dependent on the work function Ф and the field enhancement factor *β* that is related to geometric configuration of the emitter, crystal structure, conductivity, and so on.

With inherent properties of being thermally stable and oxidation resistant, ZnO nanostructures show the potential to be good candidates for field emission. Moreover, a variety of ZnO nanostructures can be synthesized by facile solution phase methods as discussed above, which not only reduce the cost, but also make it possible to fabricate flexible field emission devices based on polymers or other metal substrate materials [69,70].

**Figure 9:**
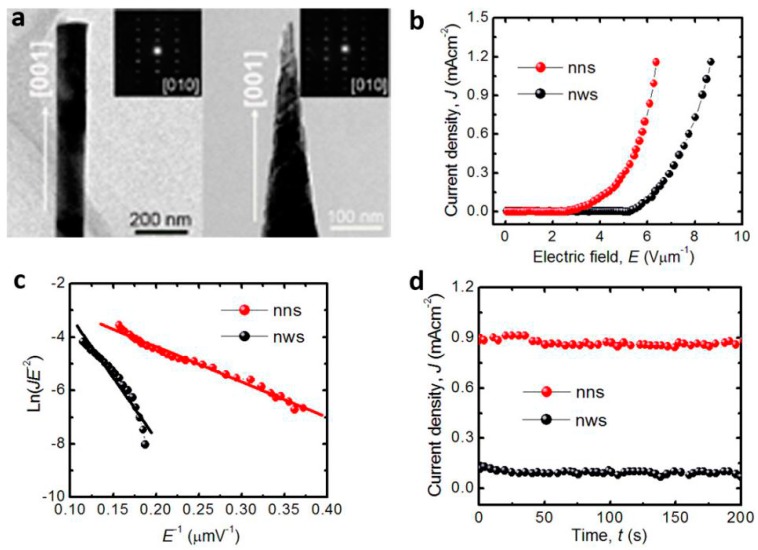
The comparison of field emission properties of ZnO nanowires with flat ends and nanoneedles with sharp ends. (**a**) Transmission electron microscopy (TEM) images; (**b**) Current density (*J*)-applied electric field (*E*) curves; (**c**) Fowler–Nordheim plots; (**d**) stability of the emission current density under a constant electric field of 6.0 V μm^−1^ (reprinted from [74] with permission, Copyright—Elsevier B.V., 2017).

Field emission studies on ZnO nanorod arrays synthesized on zinc foils by the solvothermal route are presented by Dev et al. [71]. The effect of solvothermal parameters including the solvent (distilled water and ethylenediamine), temperature, and time on the morphology and field emission properties of ZnO nanostructures are studied. It was observed that, with the increase in ethylenediamine concentrations, the alignment of the nanorods gets better, corresponding to the increase of field enhancement factor from 850 to 1044. ZnO nanotube arrays were prepared by hydrothermal reaction in ammonia and zinc chloride solutions by Wei et al. [72]. The turn-on field of the ZnO nanotube arrays was extrapolated to be about 7.0 V m^−1^ at a current density of 0.1 A cm^−2^, the emission current densities reached 1 mA cm^−2^ at a bias field of 17.8 V m^−1^, and the field enhancement factor was estimated to be 910. Cao et al. [73] reported the field emission of wafer-scale ZnO nanoneedle arrays synthesized by template-free electrochemical deposition method. The field enhancement factor of the ZnO nanoneedle arrays was 657 with a working distance of 250 μm between the cathode and anode. In our studies [74], we compared the field emission properties of ZnO nanowire arrays with flat ends and nanoneedle arrays with sharp ends (Figure 9a). The ZnO nanowires were synthesized by the hydrothermal method at 70 °C. Then, solution etching was employed to form ZnO nanoneedles at room temperature. The turn-on electronic fields of ZnO nanoneedles and nanowires are 2.7 and 5.3 V μm^−1^ at a current density of 10 μA cm^−2^. The threshold electronic fields, which were defined as the field value at the emission current density *J* of 0.1 mA cm^−2^, of ZnO nanoneedles and nanowires are 3.9 and 6.1 V μm^−1^, respectively (Figure 9b). The field enhancement factors were estimated to be 4939.3 for ZnO nanoneedles and 1423.6 for ZnO nanowires (Figure 9c). In addition, there is no obvious degradation of the current density, demonstrating the excellent emission stability of the ZnO array materials (Figure 9d). This study highlights the important effect of emitter geometry on the field emission.

Besides sample geometry, intrinsic electric conductivity in ZnO material also influences the field emission properties. Using a simple solution reduction method, oxygen-deficient ZnO nanorod arrays were synthesized by Su et al. [75]. The concentration of oxygen vacancies can be effectively controlled by adjusting the reduction temperature ranging from 30 to 110 °C, resulting in a controlled tailoring of the band structure of the ZnO. The final oxygen-deficient ZnO nanorod arrays with optimized topography show excellent field emission properties, the threshold electronic field was as low as 0.67 V μm^−1^, the field enhancement factor was as large as 64,601, and the stability was also favorable. In addition, doping with metal [76] or non-metal elements [77] through facile solution phase methods is also an effective method to improve the field emission properties of ZnO nanostructures. Doping induced conductivity enhancement and electron increase in the conduction band are the possible reasons for the emission properties improvement.

### 3.3. Electrochemical Sensors

**Figure 10:**
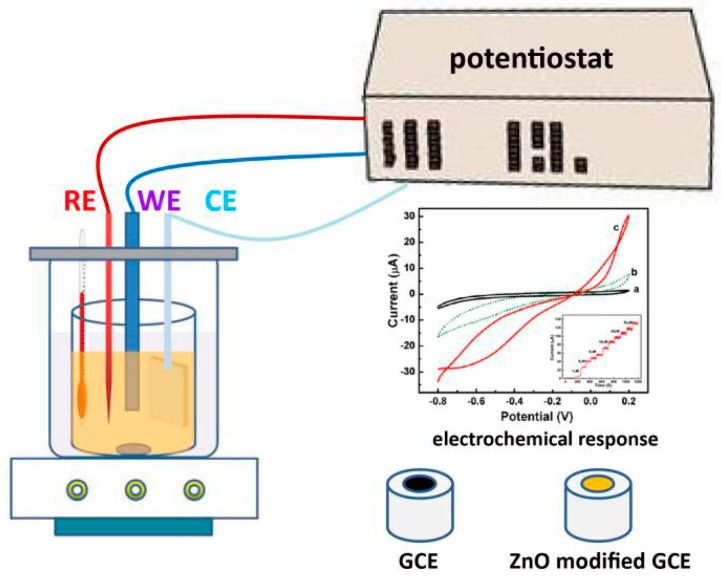
Schematic illustration of the electrochemical sensor testing.

Continuous monitoring of biological molecules and metal ions has attracted much interest due to the significant use in biotechnology, medicines, food and processing industry, and as a valuable biological marker for many oxidative biological reactions. In this regard, electrochemical sensors show unique advantages of high sensitivity, wide range of detection, real-time monitoring, ease of fabrication and control, reproducibility, and low cost, which can not be simultaneously achieved by other techniques, such as radioisotope tracing and nuclear magnetic resonance. The principle of electrochemical sensors is based on electroanalytical chemistry techniques in which quantitative investigating sensing is made by varying the potential and measuring the resulting current as an analyte reacts electrochemically with the working electrodes surface (nanostructures modified glass carbon electrode, (GCE), Figure 10). The frequently used electrochemical techniques employed in sensors include cyclic voltammetry (CV), linear scan voltammograms (LSV), differential pulse voltammetry (DPV), electrochemical impedance spectroscopy (EIS), and so on.

Thanks to the biocompatible and nontoxic nature and high sensitivity to chemical species, ZnO nanostructures have been intensively studied as different kinds of electrochemical sensors to monitor important biological molecules and metal ions in organism, typically including glucose, dopamine, uric acid, L-lactic acid, L-Cysteine, hydrogen peroxide, potassium, sodium, calcium, magnesium, and iron ions. Due to the large surface area and porous nature, 3D ZnO hierarchical nanostructures based electrochemical sensors generally show good sensitivity, low detection limit, long-term stability, and repeatability.

**Figure 11:**
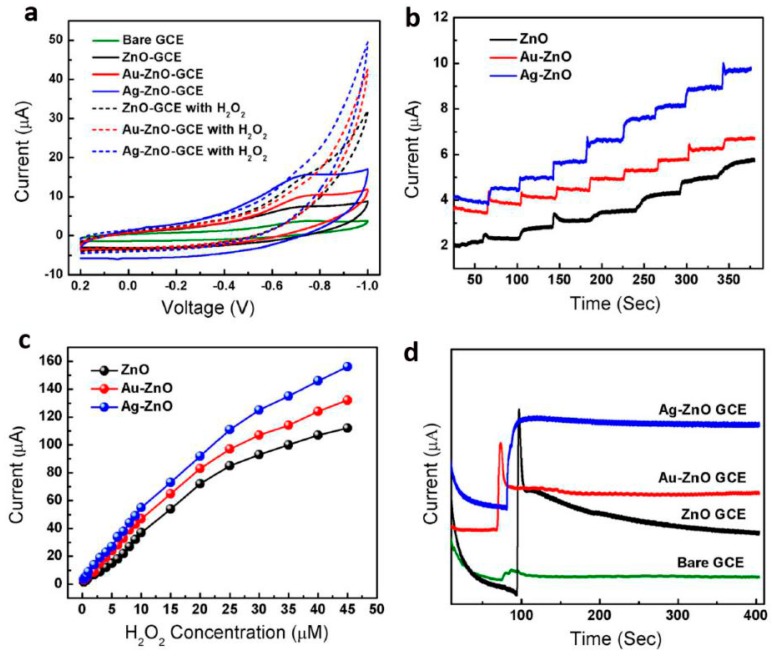
(**a**) cyclic voltammograms of bare and modified GCE with pure ZnO, Au/ZnO, and Ag/ZnO in the absence of H_2_O_2_ and in the presence of H_2_O_2_; (**b**) amperometric response of three modified GCE at constant voltage of −0.45 V with successive addition of 1 μM H_2_O_2_ in 0.05 M PBS under stirring; (**c**) corresponding calibration curves of the three modified electrodes and (**d**) stability plot of the three modified GCE at constant potential of −0.45 V in the presence of 1 μM H_2_O_2_ (reprinted from [81] with permission, Copyright—Springer Science+Business Media Dordrecht, 2016).

Yang et al. [78] prepared ordered single-crystal ZnO nanotube arrays on indium-doped tin oxide coated glass by combining electrochemical deposition and subsequent chemical etching methods. The samples were used as a working electrode to fabricate an enzyme-based glucose biosensor, which exhibited high sensitivity of 30.85 μA cm^−2^ mM^−1^ at an applied potential of +0.8 V vs. saturated calomel electrode (SCE), wide linear calibration ranges from 10 μM to 4.2 mM, and a low limit of detection at 10 μM for sensing of glucose. We studied electrochemical sensing of hydrogen peroxide by using noble metal nanoparticle-functionalized ZnO nanoflowers. Firstly, the hierarchical flower-like ZnO structures were synthesized by a co-precipitation method in a solution containing Zn (NO_3_)_2_·6H_2_O and KOH. Au or Ag nanoparticles were decorated on the surface of ZnO nanoflowers by subsequent hydrothermal treatment. Au/ZnO, Ag/ZnO and bare ZnO nanostructures modified GCE were fabricated and used as H_2_O_2_ sensors. The electrodes were tested in 0.05 M Phosphate buffered saline at pH = 7.2 with a platinum counter electrode and a saturated calomel electrode (SCE) reference electrode. CV results show that the electrochemical oxidation of H_2_O_2_ started at about −0.68 to −0.1 V versus SCE (Figure 11a), and the CV response for Ag/ZnO electrode was much higher than Au/ZnO and bars ZnO electrodes. In addition, the Ag/ZnO electrode also exhibited rapid and sensitive response to the change in concentration of H_2_O_2_ and the amperometric current is noticeably increased upon successive addition of H_2_O_2_ (Figure 11b). The linear range of calibration curve for these modified electrodes was from 1 to 20 μM (correction factor, R = −0.998) with a low limit of detection (LOD) of about −2.5 μM (Figure 11c). The sensitivity of the H_2_O_2_ sensor for Ag/ZnO modified electrode is 50.8 μA cm^−2^ μM^−1^, which is much higher than that of Au/ZnO and bare ZnO electrodes. Stability test showed that Ag/ZnO modified electrode was more stable as compared to Au/ZnO and bare ZnO showing higher value of current with steady state current loss of 1.5% after 300 s (Figure 11d). This work demonstrated that noble metal-integrated ZnO nanostructures provided a new platform for applications in designing enzymeless biosensors. By decorating ZnO nanostructures with optimized alloy clusters, the electrochemical activity can be further improved [79].

Due to the mild condition of solution phase synthesis, ZnO nanostructures can be directly grown on a wide range of flexible and conductive substrates, making it possible to fabricate free-standing and flexible electrochemical sensors. For example, ZnO nanorods were uniformly anchored on the surface of carbon cloth by a simple hydrothermal method [80]. The product was directly used as an electrode for the simultaneous determination of dihydroxybenzene isomers. The electrodes showed good electrochemical stability, high sensitivity, and high selectivity. The linear ranges of concentration for hydroquinone, catechol, and resorcinol were 2–30, 2–45, and 2–385 μM, respectively, and the corresponding limits of detection (S/N = 3) were 0.57, 0.81, and 7.2 μM.

### 3.4. Lithium Ion Batteries

With a growing world population and increasing industrialization, energy and environment become the two main factors that restrict the society sustainability. It is thus of urgent need to develop renewable energy conversion and storage techniques. Lithium ion batteries are one of the most important energy storage devices, which dominate the market of portable electronic devices, and also show potential in hybrid/electric vehicles. A typical lithium ion batteries system mainly includes anode, cathode, electrolyte, and separator, of which active materials used in both electrodes play pivotal roles in determining the overall performance of batteries. For example, the specific capacity of graphite anode in current commercial lithium ion batteries is as low as 372 mA·h·g^−1^, which is insufficient for many applications. Searching for high-performance electrode materials remains one of the most important focuses in the battery community. Among many potential electrode candidates, ZnO nanostructures have attracted much attention due to the abundance of raw materials, environmental benignity as well as facile synthesis [82,83,84,85,86]. In principle, the reaction between lithium and ZnO anodes occurs through the so-called mechanism of “conversion reaction”. During lithiation, the ZnO anode undergoes a conversion reaction to form Li_2_O embedded with nanosized metallic zinc clusters. This step is followed by an alloying reaction between lithium and the formed Zn NPs. The reaction processes are described as the following equations:

ZnO + 2Li^+^ + 2e^−^ ↔ Zn + Li_2_O (conversion step),

Zn + Li^+^ + e^−^ ↔ LiZn (alloying step),

which yields a higher theoretical capacity (987 mA·h·g^−1^) than that of graphite [87,88,89]. However, the practical use of ZnO based anodes mainly suffer from low Columbic efficiency (especially in the first cycle), severe capacity fading, and poor electrochemical kinetics [90,91]. Firstly, the conversion step in the lithiation reaction represents the largely irreversible reduction process of ZnO to metallic Zn. This irreversible chemical transformation is partly responsible for the large initial irreversible capacity loss. Secondly, the alloying step is accompanied by a large volume change (~228%) upon cycling, which results in material pulverization, electrode failure, and thus rapid capacity fading. The volume change of the anodes also results in the formation of an unstable solid-electrolyte interphase (SEI) layer, which continuously traps Li ions, leading to capacity loss. Thirdly, the low intrinsic electronic conductivity of ZnO materials causes a moderate lithium ion diffusion coefficient and limits the high-rate applications. To enhance the lithium storage properties of ZnO anodes, the construction of 3D ZnO hierarchical nanostructures with proper morphology, composition, and assembly has been proven to be an effective approach to overcome the above limitations. (1) Capacity—Compared to the corresponding nanobuilding blocks, hierarchical structures possess larger surface area, which increases the contact area between electrode and electrolyte and thus the number of active sites for electrode reactions with lithium ions. In addition, the hierarchical electrodes can lead to new lithium storage mechanisms, such as surface, interface, and nanopore storage, which lead to excess capacity. (2) Stability—The low-dimensional ZnO nanobuilding blocks have high mechanical strength, more resistance to mechanical damage, and can be engineered to allow volume change, and the assembled hierarchical structures can also prevent the possible agglomeration during the continuous cycling. Both are essential to ensure the structural integrity of the electrodes and long-term stability. (3) Rate performance—The rate of battery operation is related to the solid-state diffusion of lithium ions in the electrodes, which can be reduced in the nanoscale electrodes.

The assembly of ZnO hierarchical nanostructures shows great influence on the battery performance. Zhang et al. [92] synthesized ZnO nanostructures with different morphology by a facile hydrothermal and subsequent annealing treatment. The ZnO particles anode delivers the largest initial discharge capacity of 1815.8 mA·h·g^−1^, and a reversible charge capacity of 870.0 mA·h·g^−1^ at the current density of 50 mA·g^−1^, while cabbage-like ZnO nanosheets’ electrodes displays better cycling stability. In other work, ZnO nanorod arrays with dandelion-like morphology were grown on copper substrates by a hydrothermal synthesis method [81]. The samples can be directly used as electrodes without any additives or binders. Cycling performance was performed at a current density of 0.1 mA cm^−2^. The charge capacity of the dandelion-like ZnO electrode decreases to 596, 481 and 419 mA·h·g^−1^ in the second, third and fifth cycles, respectively. The ZnO arrays keep a capacity larger than 310 mA·h·g^−1^ even after 40 cycles, which is about four times higher than the stabilized capacity of the bulk ZnO electrode. The unique dandelion-like binary-structure played an important role in the electrochemical performance of the array electrodes.

**Figure 12:**
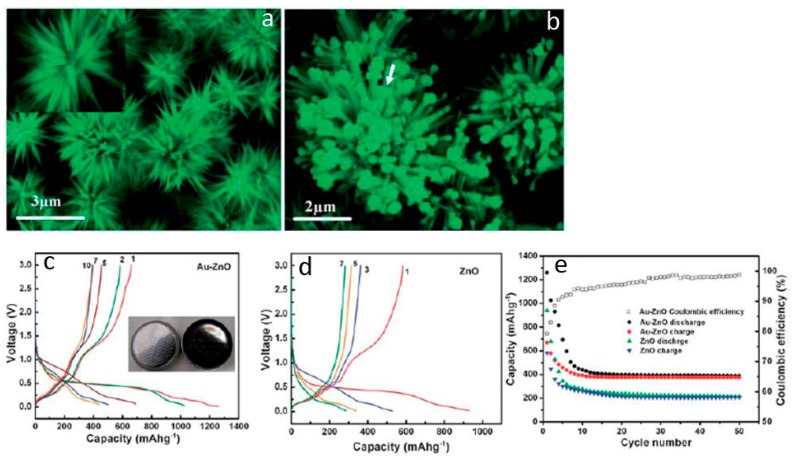
(**a**,**b**) Electron microscopy images and (**c**–**e**) lithium storage properties of the ZnO needle flowers and the Au nanoparticles/ZnO needle flowers composite (reprinted from [65] with permission, Copyright—The Royal Society of Chemistry, 2011).

Besides the architecture design, the electrochemical properties of ZnO anodes can further be improved by composting with electronically conductive agents (such as carbon nanofibers, carbon nanotubes, graphene, metals, metal compounds and so on) [93,94,95,96]. Those additives can not only enhance the conductivity of the electrodes but also modify the chemistry at the electrode/electrolyte interface. Therefore, 3D ZnO hierarchical nanostructures with suitable surface or interface composition modification show unique advantageous as improved lithium storage properties. In our previous studies, we synthesized hierarchical flower-like ZnO nanostructure by a facile solution phase approach. Au nanoparticles were functionalized on the surface of ZnO by subsequent electrochemical deposition treatment. The diameter of the pristine ZnO microflower is about 6–10 μm, and the length of an individual nanoneedle varies by 2–3 μm (Figure 12a). After electrodeposition, Au nanoparticles with an average diameter of 4–6 nm are decorated on the surface of each ZnO nanoneedle (Figure 12b,c). Comparing to the bare ZnO material, the Au-ZnO hybrid hierarchical structures possess large specific surface area, abundant void spaces, stable structure and strong electronic interaction between Au nanoparticles and ZnO. Those structural characters are beneficial for lithium storage enhancement. The initial discharge and charge capacity of Au-ZnO electrode are 1280 and 660 mA·h·g^−1^, respectively, yielding a Coulombic efficiency of 79% (Figure 12d). In comparison, the initial discharge and charge capacity of pure ZnO electrode are 958 and 590 mA·h·g^−1^, respectively (Figure 12e). The initial Coulombic efficiency of ZnO electrode is 52%, which is 27% lower than that of the Au–ZnO hybrids electrode. The stability test results show that the charge capacity of the Au–ZnO electrode decreases to 519 and 485 mA·h·g^−1^ after the second and third cycle, and stabilizes at 392 mA·h·g^−1^ after 50 cycles (Figure 12f). In contrast, the capacity of the ZnO electrode decays rapidly to 252 mA·h·g^−1^ (Figure 11f). The better lithium storage properties, including improved capacity and cycle life of the Au–ZnO electrode, can be attributed to the Au nanoparticles, which act as good electronic conductors and serve as a good catalyst during the lithiation/delithiation process. Due to the strong electronic interaction between Au nanoparticles and ZnO, electrons can easily reach all the positions where lithium ions’ intercalation takes place. This feature is very important when the battery is cycled at high current density.

**Figure 13:**
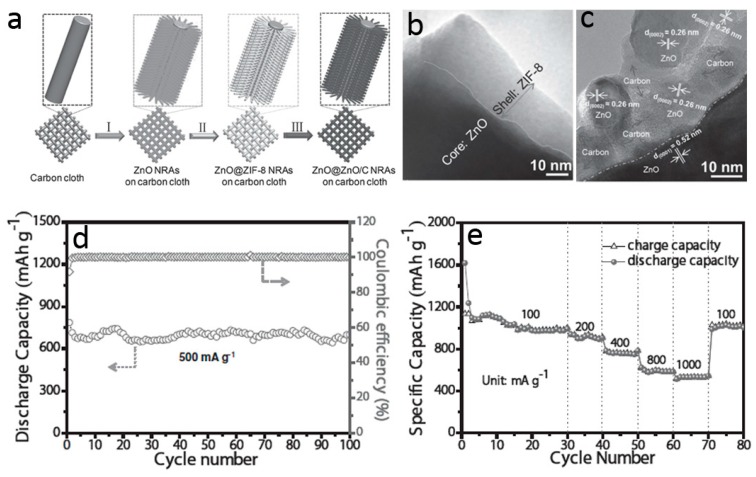
(**a**) Schematic illustrating the synthesis procedures of ZnO@ZnO QDs/C core-shell nanorod arrays on carbon cloth; (**b**,**c**) TEM and high-resolution transmission electron microscopy (HRTEM) images; (**d**,**e**) lithium storage properties of ZnO@ZnO QDs/C core-shell structures (reprinted from [100] with permission, Copyright—John Wiley and Sons, 2015).

Anchoring ZnO nanostructures on various carbon materials, such as graphite, mesoporous carbon, mesoporous carbon bubble, hierarchical porous carbon, vertically aligned graphene, and graphene aerogels, also facilitates the electron and lithium ion transport during charge/discharge cycles [97,98,99]. Those composite anodes show improved lithium storage properties, especially high rate capacity, which are highly dependent on the strong interaction between ZnO and carbon nanostructures. To strengthen this adhesion, one strategy is in situ formation carbon modification and ZnO nanostructures from metal organic compounds containing carbon and zinc elements. For example, Zhang and co-workers designed a facile and scalable strategy to synthesize integrated, binder-free, and lightweight ZnO nanoarray-based electrode as shown in Figure 13a [100]. Firstly, aligned and ordered ZnO nanorods were grown on carbon cloth via a low temperature solution deposition method. The ZnO nanorods were then served as the template as well as the Zn source to induce the formation of zeolitic imidazolate frameworks-8 (ZIF-8), a typical metal-organic framework, on the surface of ZnO nanorods (Figure 13b). Finally, unique ZnO@ZnO QDs/C core–shell nanorod arrays were obtained by thermal treatment in N_2_. Structure studies show that the shell of each nanorod is constituted by amorphous carbon framework and ultrafine ZnO quantum dots (Figure 13c), resulting in a stronger adhesive force between carbon and the active ZnO materials, which is important in order to accelerate the charge transfer in the electrode. Benefitting from this structure design, the resultant ZnO@ZnO QDs/C anode not only exhibits remarkable cycling performance (Figure 13d), but also provides a remarkable rate capability, i.e., a reversible capability of 1055, 913, 762, 591, and 530 mA·h·g^−1^ at the current density of 100, 200, 400, 800, and 1000 mA·g^−1^ (Figure 13e).

It is worth mentioning that the ZnO nanostructures can also be used as anodes for sodium ion batteries [101,102], which are important complementarities of current lithium ion batteries. Due to the fact that sodium chemistry is similar to the case of lithium, the established electrode-design strategies for ZnO materials in lithium ion batteries system can be transferred to and expedite the sodium ion battery studies.

## 4. Conclusions

In recent years, there have been explosive research and development efforts on ZnO materials, ranging from facile synthesis to advanced characterizations and device applications. 3D ZnO hierarchical nanomaterials possess a high surface area with porous structures, and facilitate multiple physical and chemical processes. In addition, the hierarchical materials not only inherit the excellent properties of an individual nanostructure but also generate new properties due to the interactions between the nano building blocks. Therefore, 3D ZnO hierarchical nanostructures provide a wide range of applications. This review article summarized the main progress in the synthesis of 3D ZnO hierarchical nanostructures via different solution phase methods, such as precipitation, microemulsions, hydrothermal/solvothermal, sol-gel, electrochemical deposition, and chemical bath deposition. In each method, the synthesis principle, factors that affect the final structure and morphology, and typical examples are briefly discussed. Then, the applications of the 3D hierarchical architectures in the fields of photocatalysis, field emission, electrochemical sensors, and lithium ion batteries are analyzed, especially the effect of hierarchical morphology on the performance are evaluated. Despite these impressive advances, several challenges still remain.

(1) Although great success has been made on the controllable synthesis of 3D ZnO hierarchical architectures, there is still room for improvement in terms of quality and scale of the products. Moreover, new synthesis methods also provide opportunities to explore novel morphology and understand the formation mechanism of the nanostructures.
(2) Besides the hierarchical architectures, the device performance is also related to the size, composition and defects of ZnO nanostructures. Coupling the 3D nanostructures with the ability of fine control over geometry and chemistry can further optimize the chemical and physical properties.
(3) Direct study of the dynamic morphological and chemical evolution of ZnO nanostructures during the practical applications is of significance to study the performance degradation and develop strategies to improve the stability.

## Figures and Tables

**Table 1:** The comparison of solution phase methods for synthesis of 3D ZnO nanostructures.

Synthesis Methods	Advantages	Disadvantages
Precipitation	Simplicity, low cost, and rapid	The nucleation and growth occur simultaneously due to the rapid reaction, making it difficult to study the detail growth process; sometimes, further thermal treatment is needed
Microemulsions	Novel morphology can be obtained by selecting suitable microemulsion system as a reactor (template)	Surfactants are difficult to remove; upscale synthesis may be hindered by the high price of some surfactants
Hydrothermal/solvothermal	Simple equipment (autoclave), low cost, and large area uniform production	Higher pressure and reaction temperature; organic solvents are needed for solvothermal method
Sol-gel	Simplicity, low cost, and relatively mild conditions of synthesis	Sol-gel matrix components may involved in the samples and additional purification is needed
Electrochemical deposition	Low synthesis temperature, low cost, and rapid; the structure and morphology can be easily controlled by electrochemical parameters	The growth substrate must be conductive
Chemical bath deposition	Simple cost, effective, and the samples can be deposited at arbitrary substrates	heterogeneous growth at the growth substrate and homogeneous formation in the bath take place at the same time; wastage of solution after every deposition
